# Policy-Led Comparative Environmental Risk Assessment of Genetically Modified Crops: Testing for Increased Risk Rather Than Profiling Phenotypes Leads to Predictable and Transparent Decision-Making

**DOI:** 10.3389/fbioe.2018.00043

**Published:** 2018-04-10

**Authors:** Alan Raybould, Phil Macdonald

**Affiliations:** ^1^Syngenta Crop Protection AG, Basel, Switzerland; ^2^Plant Health Science Services, Canadian Food Inspection Agency, Ottawa, ON, Canada

**Keywords:** risk assessment, genetically modified crops, regulatory policy, problem formulation, profiling, hypothesis testing

## Abstract

We describe two contrasting methods of comparative environmental risk assessment for genetically modified (GM) crops. Both are science-based, in the sense that they use science to help make decisions, but they differ in the relationship between science and policy. Policy-led comparative risk assessment begins by defining what would be regarded as unacceptable changes when the use a particular GM crop replaces an accepted use of another crop. Hypotheses that these changes will not occur are tested using existing or new data, and corroboration or falsification of the hypotheses is used to inform decision-making. Science-led comparative risk assessment, on the other hand, tends to test null hypotheses of no difference between a GM crop and a comparator. The variables that are compared may have little or no relevance to any previously stated policy objective and hence decision-making tends to be *ad hoc* in response to possibly spurious statistical significance. We argue that policy-led comparative risk assessment is the far more effective method. With this in mind, we caution that phenotypic profiling of GM crops, particularly with omics methods, is potentially detrimental to risk assessment.

## Introduction

Regulatory risk-management of GM crops often uses comparative risk assessment to inform decision-making. Decisions may include whether to allow cultivation or importation of a particular crop in the relevant jurisdiction, and whether any conditions need to be placed on those uses if they are permitted. Comparative risk assessment contextualizes the risk by comparing the risks posed by the cultivation of the GM crop with the risks posed by the cultivation of the non-GM counterpart. If the risk assessment indicates that cultivating a GM crop poses no greater environmental risk than cultivating the non-GM counterpart, then it might be thought that cultivating the GM crop poses no unacceptable risk. However, judging the acceptability of a risk goes beyond the scientific comparison of relative risks. In order to make this point, we discuss definitions of risk, opportunity and acceptability. We concentrate on environmental risk assessment and GM crops, but our discussion is pertinent to risk assessment and decision-making more generally.

### Defining risk and opportunity

Risk may be expressed as a combination of the likelihood and severity of harm that may arise from hazardous properties of a proposed activity. Environmental risk assessors often think of risk in terms of the potential exposure to the hazard that can cause a harm, where potential exposure is the expression of likelihood. Seriousness of harm is related to the degree of hazard, but also contains subjective elements (see below). Risk is usually difficult to quantify precisely, and most risk assessments rely on qualitative assessments and expert judgment. If severe harm is likely, risk is high; and if the most serious conceivable effect is trivial and unlikely, then risk may be regarded as negligible. However, even a tiny probability of a harmful effect may be regarded as high risk if the harmful effect is serious. A severe decline in the population size of an endangered or iconic species might be one such effect. Risk may also be regarded as non-negligible if low severity events are predicted to occur frequently (e.g., Slovic, 1999).

Similar considerations apply to the opportunities that may arise from an activity. Opportunity is high if very valuable benefits are likely to arise, such as shifts to more sustainable agricultural practices as have been seen in Canada with the widespread adoption of GM herbicide-tolerant (GMHT) canola varieties. Use of tillage by growers prior to seeding for weed control for canola appears to have been eliminated and the significant shift to minimum and zero tillage systems has reduced soil erosion, resulted in higher carbon sequestration in production areas, reduced the need for herbicide applications and created net economic benefits for growers (Gusta et al., 2011; Smythe et al., 2011). Opportunity is negligible if the most valuable benefit is unlikely and of low value, such as cultivation of a GM drought tolerant crop in an area where precipitation is almost never yield limiting. Opportunity may still be regarded as high if beneficial effects are unlikely, but would be hugely valuable if they arose. The reduction of a non-target effect to a highly beneficial or iconic insect species that may only rarely co-occur with crop production could be considered as highly beneficial. This may occur if cultivation of the GM crop reduces the spraying of pesticides, either directly through endogenous insect protection or indirectly by carrying a disease tolerance that reduces the need to spray for an insect vector of the disease. Significant opportunity may also accrue from frequent events of relatively low value.

### Judging the acceptability of risk

Judging the acceptability of risk requires a method to weigh the opportunities against the risks of the activity under consideration (Sanvido et al., 2012). Under ethical decision-making, if a risk exceeds an acceptability threshold, then the risk is unacceptable regardless of the size of the opportunity. Under utilitarian decision-making, the course of action posing the highest net opportunity—the opportunity minus the risk—must be selected. It follows that even severe risks may be acceptable provided the opportunities are high enough, and that an increase in risk many be acceptable provided it is outweighed by increased opportunity.

In practice, determining the acceptability of risk for the cultivation of a GM crop is made difficult by the need to balance complicated sector needs with a broader public good. The 1993 Canadian Regulatory Framework for Biotechnology (Industry Canada, 1998; Gabler, 2008), for example, attempts to articulate guiding principles for how decisions could be structured. The framework captures the idea that any regulatory decisions should enable innovation, but also protect the environment and the health and well-being of citizens. Governments often have competing internal interests where departments of environment may view the opportunities for cultivating GM crops differently from Departments of Agriculture who see the acceptable risks and benefits of agriculture with a more commercial perspective.

Determining whether an activity poses acceptable risk requires several difficult judgments. First, one must decide what would be regarded as harmful effects of the activity and what would be regarded as beneficial effects. In addition, one must decide how to judge the severity of harm and the value of benefits. While science may be used to limit the scope of discussions of harm and benefit to plausible effects of the proposed activity (Raybould, 2010a), the designation of an effect as harmful, beneficial or neither, and the severity and value ascribed respectively to harmful and beneficial effects of a particular size relies on non-scientific criteria. These criteria may be based on personal values, an organization's objectives or public policy depending on who will make the decision. For brevity, hereafter we refer to these non-scientific criteria as “policy objectives.”

The second difficult judgment is how one will weigh risk and opportunity. One must consider whether certain effects should be unacceptable regardless of the size of the opportunity or whether the largest net opportunity will always be the preferred option. In addition, one will need a method for evaluating net opportunity when benefits and harms may be very different; how, for example, does one evaluate the net opportunity if growing a certain crop is expected to increase yield but reduce other ecosystem services (de Groot et al., 2010).

**Figure 1:**
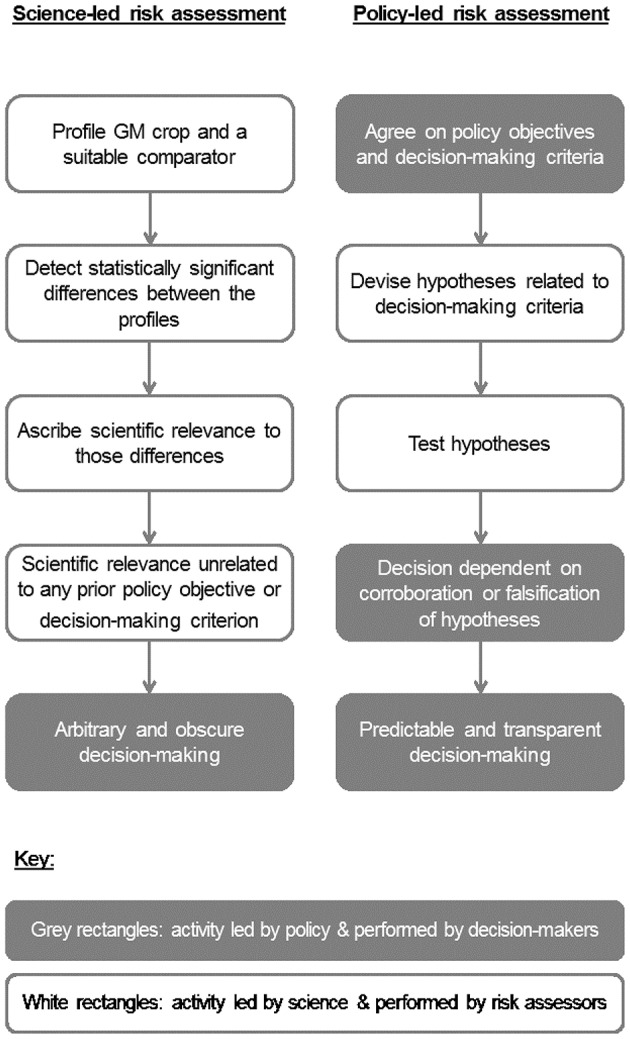
Conceptual models of science-led and policy-led risk assessment.

The above considerations show the importance of setting clear policy objectives in order to ensure that the scientific parts of risk assessment answer questions that are useful for decision-makers rather than questions that scientists may find interesting (Hill and Sendashonga, 2003; Evans et al., 2006). In practice, even with policy direction, such as a policy objective on the conservation of biodiversity, risk assessors rely on professional judgment when they weigh evidence in what is often a qualitative process and make a number of “micro policy judgments” while conducting the assessment. Indeed, the promotion of “science-based risk assessment” (= science-led in our terms) (e.g., Andow and Hilbeck, 2004; Kuntz et al., 2013) could lead to the mistaken and pernicious idea that it is desirable to eliminate consideration of policy objectives and judgment from risk assessment. Such thinking is almost guaranteed to produce controversy and paralyze decision-making (e.g., Raybould, 2010b). Instead, “policy-led risk assessment” ought to be the aim (Figure 1).

In this article, we explore the implications of a change of emphasis from science to policy on two aspects of comparative environmental risk assessment of GM crops that are of current interest: problem formulation and the use of profiling data from various omics techniques. While we focus on regulatory decision-making about GM crops, our remarks are relevant to all crops with novel phenotypes, however they are produced, and to other types of decision-making, such as choosing which products to develop (Macdonald, 2014).

## Problem formulation

### Risk hypotheses and decision-making criteria

In essence, regulatory risk assessments should test hypotheses that help risk managers to make good decisions about whether to permit particular activities. Problem formulation is the process by which these risk hypotheses, and plans to test them, are devised. While we concentrate on environmental risk posed by the cultivation of GM crops, our comments are relevant to any regulatory decision-making that makes use of risk assessment.

In regulatory environmental risk assessment, decision-making criteria should relate to the probability and severity of environmentally harmful effects arising from the proposed activity covered by the regulations. In the case of GM crops, the proposed activity will be cultivation of a specified GM crop in a particular place, perhaps with other stipulations such as whether certain crop-protection chemicals will be applied to the crop. The definition of what is harmful is a matter for the risk managers based on their interpretation of the policy objectives of the legislation that the regulations are designed to implement.

At their most conservative, the risk hypotheses will be that no harmful effect will result from the proposed activity. If these hypotheses are corroborated under rigorous testing using information from reputable sources, including data from laboratory or field tests, the risk managers can be confident that the proposed activity poses negligible risk, and then use that conclusion in their decision-making. Less conservative risk hypotheses acknowledge the probability and contextualize the impact of any harmful effect; that is, the hypotheses under test would be that the risk does not exceed a threshold of acceptability. The threshold may be set to be the same as the risk posed by similar activities, or higher risk could be tolerated if the activity provides greater opportunities; for example, greater risk might be acceptable for cultivation of a GM crop that provides higher yield or improved quality than the crops it will replace. Rigorous corroboration of the hypotheses would indicate that the risks could be placed in the context of those from comparable activities, such as the cultivation of a non–GM crop that has a similar trait, even though the risks may not be negligible. That conclusion would contribute to decision-making.

### Placing risks in context of current practice

In theory, regulations could specify that certain effects are harmful if they are caused by the cultivation of GM crops but are not harmful if caused by other activities. However, such definitions of harm would violate accepted standards of good regulatory practice. The OECD (2014) describes eight Principles of Regulation, and defining effects as harmful only if they are caused by GM crops would violate at least three of them: Principle 2 that regulations must have a sound legal and empirical basis; Principle 4 that regulations must minimize market distortions; and Principle 7 that regulations should be consistent with other regulations and policies. Hence, definitions of acceptable risk for GM crops should consider what is regarded as acceptable for other agricultural practices.

Many publications have concluded that conceivable harmful environmental effects from cultivating GM crops are of the same type as those from growing non-GM crops (e.g., Tiedje et al., 1989; NRC, 2002; Perry et al., 2004; Lemaux, 2009). Hence, a hypothesis that growing a certain GM crop will cause no harm, is really a hypothesis that growing the GM crop will cause no greater harm than the current practice that cultivation of the GM crop may replace. Similarly, a hypothesis that growing a certain GM crop will poses no unacceptable risk, is really a hypothesis that any increase in risk caused by growing the GM crop will be acceptable, either because the increase falls below a threshold of acceptability or because the additional opportunities created by growing the crop are worth the risk. As “no additional harm” sets a higher standard than “no unacceptable increase in risk,” testing a hypothesis of no additional harm may be regarded as rigorous testing of a hypothesis of no unacceptable increase in risk provided other factors that determine acceptability of risk, such as the size of the opportunity, are unchanged.

A hypothesis that growing a GM crop will cause no unacceptable increase in risk is useful in a least three respects. First, corroboration or falsification of this hypothesis is valuable to risk managers. Second, it shows that GM regulation follows the Principles of Regulation by not treating GM crops differently from other agricultural practices. Finally, it is useful to risk assessors, because if “unacceptable risk” is sufficiently operationalized, risk assessors have clarity about the data they need in order to conduct the risk assessment, namely data that test the hypothesis of no unacceptable risk.

Consider a proposal to cultivate a new variety of GMHT canola that is likely to replace long-standing cultivation of a non-GM (“conventional”) canola. Also, suppose that the effects of recommended herbicide applications to the GMHT canola fall under regulations covering GM crops and the effects of recommended herbicide application to the conventional canola are covered by pesticide regulations. A possible effect of switching from conventional canola to the GMHT canola is a change in the abundance and species diversity of weeds owing to variation in their sensitivity to the different herbicides used on these crops (e.g., Perry et al., 2004; Wilson et al., 2007). In assessing the risks posed by cultivating the GMHT canola, the Principles of Regulation suggest that it would be unreasonable to compare the weed flora in the GMHT canola regime with the weed flora if no herbicides were used; the comparison ought to be with the conventional herbicide management.

### Assessing risks rather than measuring differences

Identifying a fair comparator is only a partial solution to the problem of formulating a useful risk hypothesis. Countless changes in the weed flora are theoretically possible when switching from conventional to GMHT weed management. Science-led risk assessment (Figure 1) might approach this problem by setting up multiple field trials at many sites over many years to measure the change in the weed flora when GMHT replaces conventional management; in effect, the hypothesis under test would be one of no difference between the weed floras of conventional and GMHT canola.

Comparing weed diversity and abundance between conventional and GMHT canola will almost inevitably reveal numerous statistically significant differences (e.g., Heard et al., 2003a,b), with the number limited only by the size of the experiments, the sensitivity of the measuring techniques and the imaginations of the researchers in devising ways to categorize difference. However, few or even none of these differences may have any relevance to regulatory policy objectives. Consequently, cataloging differences is at best an inefficient way to conduct risk assessment, because effort is wasted on measurements of no value for decision-making. At worst it is ineffective and potentially counterproductive because decisions are made *ad hoc* in response to statistical significance, which can easily be spurious when many variables are measured (Benjamini and Hochberg, 1995; Leek et al., 2017), rather than after serious consideration of what the objectives of agricultural and environmental policies ought to be. We could call this behavior PARKing—*P*olicymaking *A*fter the *R*esults are *K*nown—based on Kerr's (1998) term HARKing for *H*ypothesizing *A*fter the *R*esults are *K*nown.

Policy-led risk assessment would approach the problem by defining, at the very least, general trends that would be regarded as harmful changes in the weed flora; harmful meaning detrimental to achieving policy objectives. One might define harm of cultivating the GMHT canola as an increase in the abundance of specific species of economically damaging weeds, or a decrease in abundance of specific species that may have aesthetic or nature-conservation value, compared with their abundance under conventional management (e.g., Pimentel et al., 2001). Another option would be the incorporation of some decision-making criteria into the definitions; thus, one might define the threshold of unacceptable harm as a 50% increase in the abundance of noxious weed X or as a 25% decrease in the abundance of endangered species Y.

Prior definition of decision-making criteria means that experiments can be designed to rigorously test risk hypotheses. One could envisage, for example, testing a hypothesis that the abundance of noxious weed X will not increase by more than 50% by testing a hypothesis that it is at least as sensitive to the herbicide that will be applied to the GMHT canola as it is to the herbicides applied to conventional canola. Such a targeted test of a policy-relevant hypothesis would be entail vastly more efficient and effective parameters for data collection than would untargeted comparisons of the weed floras of GMHT and conventional canola.

With best practices, risk assessors will contextualize the risks for cultivating the GMHT canola and compare that with the harm from the cultivation of conventional canola. In the risk assessment, the risk assessor will consider that cultivation of a monoculture and the management of a crop in an agricultural production system reduces biodiversity and has an impact on the environment. The crop plant itself has a suite of traits that result in the production of compounds that create environmental effects and influence ecosystem services. In the comparative risk assessment, the risk assessor will evaluate the relative impacts of the two phenotypes and evaluate whether the addition of the new trait creates harms that exceed those already imposed by the cultivation of the existing crop. In this scenario, the evaluation does not insist the results of growing the two crops be identical, only that the probability or severity of a harm is not increased.

Policy-led risk assessment can target risk management to make interventions in order to realize benefits and reduce harms. In testing the risk hypothesis that the endangered species Y will not decrease by more than 25%, testing may reveal that the species is more sensitive to the GMHT herbicide than to the conventional canola herbicide. This finding could trigger a search for changes to management techniques that ensure weeds are still adequately controlled while minimizing exposure of species Y to the herbicide, perhaps by altering the proposed timing, rate or method of its application (e.g., Thompson et al., 1991). In contrast, unfocussed risk assessment may reveal potential changes in the abundance of numerous species without any attempt to contextualize the risk. Faced with such a finding, risk managers may simply refuse to approve the GMHT canola (Sanvido et al., 2011), thereby foregoing opportunities and not necessarily reducing risk—although they may have reduced the probability of change.

In summary, problem formulation for comparative risk assessment of GM crops should consider two important elements. First, the comparison should be consistent with the Principles of Regulation. The effects of using the GM crop should be compared with agricultural practices that these uses will replace. Second, the selection of the hypotheses to be tested in the risk assessment should always be policy-led and informed by science. Policy-led risk assessment will guide risk assessors to develop hypotheses of known relevance to the final regulatory decision and suggest experiments that are required to improve decision-making rather than satisfying scientific curiosity. The combination of hypotheses based on prior agreement of decision-making criteria and rigorous testing maximizes the chances that risk managers will make decisions that fulfill agricultural and environmental policy objectives. Risk communication will also be improved. Science-led risk assessment, on the other hand, leads to PARKing: *ad hoc* decision-making based on whatever differences happen to reach statistical significance in comparisons of many variables. These decisions are unlikely to meet wider policy objectives. They are also likely to create controversy because decisions appear to be fixed by selecting particular data rather than after a debate about what the objectives of policy ought to be (e.g., Sarewitz, 2004).

## Profiling in risk assessment

In the example above, we proposed that rigorous testing of targeted hypotheses is a more efficient and effective approach to risk assessment than are untargeted tests of null hypotheses of no difference between a GM and a non-GM cropping system. The latter approach makes use of profiling—the characterization of a system by describing a combination of many of its attributes.

### Historic and current use of profiling in risk assessment

Profiling of GM crops is used widely in risk assessment. Compositional analysis typically tests for statistically significant differences between the GM crop and a near-isogenic comparator variety in the amounts of 60–80 nutrients and anti-nutrients (Herman and Price, 2013). Phenotypic characterization compares 30 or more aspects of germination, plant growth and development, morphology, reproduction, disease and pest damage, and attributes of grain or fiber quality depending on the crop (Horak et al., 2007). The aim of these studies is to identify differences between the GM crop and its comparator that need further evaluation in order to characterize risk to human and animal health and to the environment from using the GM crop (Kuiper et al., 2001; Nap et al., 2003).

Although not routinely required for regulatory testing, profiling of GM crops can also be carried out at the molecular level, using transcriptomics, proteomics or metabolomics (Kuiper et al., 2003). The value of these methods, along with characterization of the epigenome, for crop improvement has recently been discussed by the National Academies of Sciences, Engineering and Medicine (NAS, 2016). Our purpose here is not to evaluate the technical feasibility of molecular profiling, but to discuss whether profiling approaches generally are valuable in risk assessment of GM crops.

A claimed advantage of profiling methods is that they are unbiased (Kuiper et al., 2003). They make no assumptions about how the GM crop might differ from its non-GM counterpart. In addition, unbiased approaches make no judgment about what differences might be important in indicating that using the GM crop may pose greater risk than similar uses of the comparator. Hence, profiling approaches are science-led evaluations of potential differences with all the problems that entails (Figure 1).

In the early days of GM crop development, there was significant uncertainty about the extent to which transformation of plants could lead to unintended changes. Hence, compositional and phenotypic profiling of GM crops made sense as methods to explore the extent of these changes: testing the hypothesis that transformation introduces no unintended changes was a useful tool for basic research into the effects of transgenesis and also for risk assessors struggling to characterize products of new technology.

In retrospect, however, there was always a need to ensure that these studies were placed in context when used to inform the risk assessment. In practice, this has generally been the case when a GM crop and its non-modified counterpart are compared. For example, as changes in the nutritional value of a crop could be harmful to human and animal health, the risk assessor determines whether the amounts of key nutritional components are statistically different between the GM and non-GM comparator. If statistically significant differences are identified, the assessor will ask whether the amounts in the GM crop fall into the normal range for that crop. If they do, the differences will generally be disregarded.

It is important to recognize that comparing nutrients is policy-led risk assessment because protecting human and animal health is a policy objective. To keep the risk assessment policy-led, however, it is important that the substances tested really are determinates of health. If the most extreme conceivable change in the amount of a substance would have no material effect on health, then that substance should be of no concern for policy-led risk assessment, and comparing its concentration in the GM and non-GM crop should not be necessary to determine risk.

Without prior definitions of important changes, science-led profiling can encourage the idea that producing more data inevitably leads to better risk assessment. Statistically non-significant comparisons of thousands of substances may appear to be a more convincing demonstration of negligible risk than is the lack of difference in a few key nutrients. However, unless it is possible to specify values of particular variables that would show a policy-led risk hypothesis to be false, the data are of no relevance for drawing conclusions about risk. Finally, profiling may also understate the importance of policy in risk assessment and decision-making. It seems to promote the idea that if sufficient data are collected, uncertainty will be diminished and the “correct” policy toward the use of GMOs will become obvious.

### Profiling using omics methods

The introduction of molecular profiling methods into regulatory risk assessments would only increase the pervasiveness of unfocussed data generation rather than policy-led attitudes to risk assessment. Additional data generation will often pose questions for which there are no ready answers leading to a continuing need to produce yet more data. The ability to find differences between a GM crop and its non-GM comparator is virtually limitless, creating endless opportunities for PARKing. Advocates of molecular profiling may argue that the methods could show that variation between GM and non-GM plants as a class is insignificant compared with variation among non-GM plants. However, this misses the point. The purpose of regulatory risk assessment is not to make general points about a technology or class of products, it is to evaluate whether the risks posed by a specific use of a specific product are acceptable. Acceptability of risk is ultimately a policy decision, and anything that promotes policymaking as an *ad hoc* response to possibly spurious statistically significant differences, rather than careful deliberation about delivering agreed societal objectives, should be discouraged.

Finally, our point is not that omics methods can never have value in regulatory risk assessment. If measurements of specific transcripts, proteins or metabolites are a good test of a hypothesis that a given use of a given GM crop does not pose an unacceptable increase in risk, then the measurements may have value for regulatory decision-making. However, using the methods simply to create profiles will be a serious impediment to moving from science-led to policy-led risk assessment and decision-making.

## Conclusions

Comparative risk assessment is a valuable method for making risk assessment tractable, provided that it is policy-led rather than science-led. Ideally, policy-led comparative risk assessment for a GM crop would define effects that comprise unacceptable increases in risk from its use. The comparison would be with the acceptable effects of a similar crop in a similar agricultural system that is likely to be replaced by use of the GM crop.

Defining an unacceptable increase in risk enables the formulation of testable hypotheses for risk assessment. At their most conservative, the hypotheses will be that certain effects are no more likely to occur, and if they do occur, are no more severe than those caused by use of the crop that will be replaced. Only data that test such hypotheses, that is, are able to show them to be false, are useful for such policy-led risk assessment.

The alternative method of comparative risk assessment dispenses with policy objectives and makes numerous tests of the null hypothesis that the GM crop does not differ from the crop that it will replace. Such “science-led” risk assessment makes no judgment about the importance of the variables being measured. Proponents of this method of risk assessment see this unbiased nature of the risk assessment as a strength (e.g., Kuiper et al., 2003).

However, while lack of bias in testing a hypothesis is a virtue in risk assessment, as in all basic and applied science, lack of bias in selecting the hypotheses to be tested is a grave weakness: we should be strongly biased toward hypotheses that help decision-making and realization of policy objectives. Without this bias, policy may be formulated in response to trivial differences, perhaps influenced by ill-informed indignation that a GM crop, unsurprisingly, differs from a non-GM comparator in some respect. It is this very lack of bias that we believe makes science-led risk assessment vastly less effective than the policy-led alternative.

In advocating policy-led risk assessment, we do not underestimate the difficulties agreeing on policy objectives. Disagreement about what comprise beneficial or harmful effects of using certain GM crops is rife, even within organizations that develop and regulate them. However, sooner or later policy objectives have to be set in order to make decision-making feasible and hence risk assessment efficient and effective. While defining these objectives may be controversial, such controversy is likely to be less than that produced by making policy *ad hoc* in response to possibly spurious statistically significant differences identified by untargeted profiling methods. Ultimately, decision-makers have to decide based on their individual or organizational policy objectives. This responsibility cannot be outsourced to statistical algorithms processing vast amounts of profiling data.

## Author contributions

All authors listed have made a substantial, direct and intellectual contribution to the work, and approved it for publication.

### Conflict of interest statement

During the writing of this paper AR was employed by Syngenta and PM was employed by the Canadian Food Inspection Agency.
